# Organic amendments to potato soils inconsistently enrich yield-associated soil microbiota across growing regions of the continental US

**DOI:** 10.7717/peerj.20595

**Published:** 2026-01-29

**Authors:** Scott A. Klasek, James E. Crants, Kenneth E. Frost, Brenda K. Schroeder, Carl J. Rosen, Linda L. Kinkel

**Affiliations:** 1 Department of Plant Pathology, University of Minnesota, St. Paul, MN, United States of America; 2 Department of Soil, Water, and Climate, University of Minnesota, St. Paul, MN, United States of America; 3 Department of Botany and Plant Pathology and Hermiston Agricultural Research and Extension Center, Oregon State University, Hermiston, OR, United States of America; 4 Department of Entomology, Plant Pathology, and Nematology, University of Idaho, Moscow, ID, United States of America

**Keywords:** Microbiome, Soil, Potato, Organic amendment, Yield, Fumigation, Mustard

## Abstract

Plant health is regulated by complex consortia of soil microbes with growth-promoting and pathogenic functions. In potato production, various soil management practices are undertaken to boost yields and suppress diseases, but connections between these practices, soil microbiomes, and tuber yields have not been characterized across diverse growing regions. To identify growing practices and microbes associated with increased yields, we established four-year field trials across eight US sites from Oregon to Maine that consisted of controls, fumigations, organic amendments, and mustard incorporations. Amplicon sequencing of 16S ribosomal RNA (rRNA) genes and intergenic transcribed spacer (ITS) regions was used to investigate bacterial and eukaryotic soil microbiomes, respectively. Fumigation and organic amendment treatments increased tuber yields in 23% and 29% of treatments relative to controls. While soil treatments influenced both microbiome types differently across all field sites, eukaryotes were more sensitive than bacteria to all treatments. Across field sites, soil treatments impacted relative abundances of amplicon sequence variants (ASVs) to varying degrees, even among ASVs belonging to the same genus. Associations between ASVs and tuber yields similarly varied within genera, highlighting the lack of consistent yield-associated taxa across US growing regions. Nevertheless, forty-five “target ASVs” across nine bacterial and three fungal phyla were identified as both treatment-impacted and yield-associated within any particular field site. Models identified three of thirteen organic amendment scenarios and one of thirteen fumigation scenarios where increased relative abundances of specific target ASVs accounted for up to a 23% increase in tuber yields compared to control treatments. These ASVs were largely site-specific and not influenced by treatment-associated changes in soil nutrients or organic matter, highlighting complex relationships within field sites that require further study to achieve the goal of implementing sustainable, microbiome-informed potato production techniques.

## Introduction

Plants interact with a diversity of soil microbes that play numerous roles in plant fitness, including facilitating nutrient uptake, competing against pathogens, and stimulating host growth through hormonal signaling (Trivedi et al., 2020). Changes in plant-associated microbiomes can offer mechanistic insight into these beneficial functions, within the context of plant-microbe-environment interactions (Yu et al., 2021; Santos-Medellín et al., 2021). For these reasons, understanding, applying, and engineering the presence or abundance of specific microbes—and more broadly, the microbiomes they inhabit—has tremendous potential to deliver sustainable agricultural innovations (Compant et al., 2024; Petrushin, Filinova & Gutnik, 2024).

Potatoes are the fifth-most grown crop in the world by yield (United Nations, 2023) and play a critical role for global food security based on their high nutrient density and increased cultivation in developing areas (Devaux, Kromann & Ortiz, 2014). Because large-scale cultivation requires intensive soil disturbance and often relies on chemical fumigants to suppress soilborne pathogens, alternative strategies that improve soil health while maintaining productivity are of particular interest to growers (Larkin, 2022; Hills et al., 2020). One particular example is amending soil with organic matter, often manure or compost. This can enhance soil health by delivering nutrients, improving soil physical properties, increasing soil microbial biomass (Luo et al., 2018), altering microbial community composition, suppressing pathogens (Hao & Ashley, 2021), and even reducing agricultural greenhouse gas emissions (Brenzinger et al., 2021). The incorporation of green manures, such as mustards, into soil within potato rotations is another strategy that can reduce soilborne diseases such as Verticillium wilt, black scurf, and common scab; these are thought to act by stimulating pathogen inhibitory activity in soil microbial populations (Larkin, 2022; Larkin, Honeycutt & Olanya, 2011; Wiggins & Kinkel, 2005).

Recent work has identified members of seed tuber microbiomes that are robustly predictive of plant vigor across growing seasons (Song et al., 2025), and linked crop rotations and organic matter enrichments with tuber quality and specific microbial taxa (Hemkemeyer et al., 2024). Experimental studies have documented a wide range of functions by which taxonomically diverse microbiota promote plant growth in potato soils and rhizospheres, including nitrogen fixation, phytohormone synthesis, oxidative stress protection, and suppression of pathogens such as *Streptomyces scabei* through antibiotic production (Schlatter et al., 2017; Batool et al., 2020; Naqqash et al., 2016). Despite these efforts, how soil treatments used in potato cultivation affect relationships between soil bacterial and fungal microbiomes and tuber yields has not been established, particularly across diverse growing regions. Potato soil microbiome composition throughout growing regions of the continental US is characterized by high site-specific variation, which coincides largely with soil physicochemical properties (Klasek et al., 2023).

To examine whether soil treatments consistently enrich microbiome taxa (amplicon sequences or ASVs) across different US regions of potato production, we fumigated soils, added organic amendments, and/or incorporated mustard cover crops across eight US field sites in a four-year field trial. In the final year of the trial, we collected soil chemical and microbiome data, as well as tuber yields. We hypothesized (1) that these soil treatments would explain different proportions of variance in soil microbiome composition across field sites and taxonomic domains, and (2) that ASVs impacted by soil treatments could account for increases in tuber yields in field-specific ways. Using structural equation modeling, we identified relationships between organic matter addition, soil chemistry, microbial consortia, and tuber yields. This study provides a benchmark for growers looking to establish sustainable potato production methods and serves as a starting point for future studies linking cultivation practices (particularly organic amendments) with growth-associated or disease-suppressive members of soil microbiomes.

## Methods

Portions of this text were previously published as part of a preprint (Klasek et al., 2025).

### Experimental design

**Figure 1:**
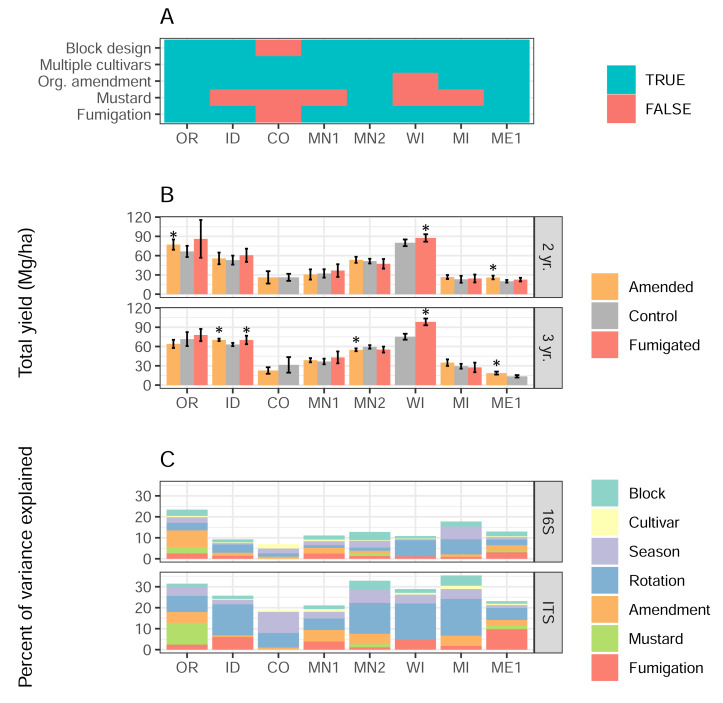
Soil treatments influence tuber yields and microbiome structure. (A) Presence/absence matrix of factors and treatments used in each field site. (B) Total yields, in Mg/hectare, of the most commonly-grown cultivar in each field site, separated by soil treatment, site, and rotation length. Error bars indicate 95% confidence intervals, and asterisks show significant differences (*p* < 0.05) between amended or fumigated treatments relative to controls. (C) Percentages of variance in 16S or ITS microbiome structure attributed to each treatment or factor of the experimental design.

Potatoes were grown in two- and three-year rotations from 2019–2022 in eight field sites representing potato growing regions across the continental US, from Oregon to Maine (map and descriptions of site locations in Klasek et al., 2023). These included six university field sites (OR, CO, MN1, WI, MI, and ME1), one private research site (ID, Miller Research LLC), and one grower field (MN2, R.D. Offutt Farms) according to US state abbreviations. Sampling and publication of results were approved by RD Offutt Farms (Fargo, ND, USA), and Miller Research LLC (Rupert, ID, USA). Every field site contained six soil and cover cropping treatments corresponding to each rotation length, with either four or five replicate plots per treatment. Plots in all fields except CO were arranged in a randomized block design; CO was arranged in a completely randomized design. For the purposes of our analyses, soil treatments were classified as amended with composted or aged manure, conventionally fumigated with metam sodium or chloropicrin, or neither (controls). Treatments were also classified based on whether a mustard green manure was tilled back into the soil as a biofumigant at any point in the four-year sequence. Organic amendments were applied before planting, while fumigants were applied in the preceding fall seasons using shank injection at 15–23 cm depth, consistent with commercial practices. Treatments were considered mutually exclusive, except for two irregularities: manure amendments were added to fumigated plots in MI, and manure amendments were confounded with mustard incorporation in MN1. Local growing regions and practices guided total N inputs, and in most cases, as the choice of cultivars and cover crops. The presence or absence of each treatment type across each field site, as well as the use of a block design and multiple potato varieties, is shown in Fig. 1A (present = true, absent = false). Cover crops varied across field sites, rotations, and treatments; in some cases they were tilled into soils as green manures, but the specific types, sequences, and green manure status of cover crops were not coded into distinct treatment categories. We defined a total of thirty-three *management scenarios*, each consisting of a soil treatment implemented at a field site under a two- or three-year rotation that corresponded to control treatments growing the same potato cultivar. More detailed treatment information, as well as four-year cropping histories, cultivars grown, rates, types, and timing of organic matter and fumigants applied, are provided for all field sites in Table S1.

### Soil sampling, DNA extraction, amplicon sequencing, and sequence processing

Potato production soils were sampled from eight field sites in 2022 at two seasonal timepoints: at planting, and 60 days after planting to coincide with maximum root growth and microbial activity. Each site consisted of twelve treatment and rotation length combinations, and four (ID, OR) or five replicate plots (all others), resulting in 912 soil samples collected from 456 plots. A 2.5-cm-diameter core was used to sample to a depth of 15 cm from locations at least 1 m from the border of each plot. For samples taken 60 days after planting, the tops and shoulders of rows and furrows were sampled. Forty cores per plot were collected in Ziploc bags, stored at 4 °C, mixed in a sterile bowl, and filtered through a 4.4-mm sieve within 48 h of collection. DNA was extracted from 0.25 g of bulk soil using Qiagen DNeasy PowerSoil Pro kits following manufacturer instructions and stored at −80 °C until shipment on dry ice to the University of Minnesota Genomics Center for amplification and sequencing.

A dual-index library preparation and sequencing approach developed to minimize amplification and primer mismatch biases was used to amplify 16S rRNA gene and ITS sequences (Gohl et al., 2016). Using 20 amplification cycles, the V3V4 region of the 16S rRNA gene was targeted with the 341F primer CCTACGGGAGGCAGCAG and the 806R primer GGACTACHVGGGTWTCTAAT. The ITS2 region was amplified with the ITS3 forward primer TC GATGAAGAACGCAGCG within the 5.8S subunit and the ITS4 reverse primer TCCTCCGCTTATTGATATGC within the large 28S subunit (Bokulich & Mills, 2013). Amplicon libraries were sequenced on an Illumina MiSeq using a 600 cycle v3 kit (2 × 300 bp reads). All libraries were partitioned across three separate runs to minimize run-specific variation (Song et al., 2018) and re-pooled.

Cutadapt v1.18 (Martin, 2011) was used to trim off portions of ITS reads that continued into primer regions of their pairs. Using R (v4.3.2), fastq reads were denoised into amplicon sequence variants (ASVs) with the DADA2 package (Callahan et al., 2016) and taxonomies were assigned with the assignTaxonomy function in DADA2 using either v138 of the SILVA nonredundant 16S SSU Ref NR99 database (Quast et al., 2013) or v8.3 of the UNITE ITS database (Kõljalg et al., 2013; Nilsson et al., 2019). The 16S sequences identified as chloroplasts, mitochondria, or eukaryotes were removed, as were the ITS sequences belonging to flowering plants (*Anthophyta*) or unassigned at the kingdom level. In eukaryotic microbiomes, 89% of reads were classified to fungi. ASV count tables, taxonomy annotations, and sample data were combined into objects using phyloseq v1.46.0 (McMurdie & Holmes, 2013). Libraries of either amplicon with less than 10,000 sequences were discarded, leaving 1,630 microbiome libraries out of the possible 1,824 (912 soil samples × 16S or ITS). All steps from soil sampling to amplicon sequence processing were performed identically to those described previously (Klasek et al., 2023).

### Tuber yields

At the end of the growing season, vines were left to die naturally (ID), killed mechanically (MN1), or killed chemically with one or two applications of diquat (two pints/acre, all other sites). If repeated, diquat was applied between one and two weeks apart. At least one week after vine death, a total of two rows within each plot (ranging from five to nine m) were harvested by machine from areas that had not been soil sampled earlier in the season. Tubers were weighed and yields were estimated based on row widths (0.86–0.91 m). For each site and rotation, two-sided t-tests (alpha = 0.05) were used to determine if yields varied between amended or fumigated treatments relative to controls, with the exception of MI, where amended treatments were compared to fumigated ones because both underwent fumigation. The most common cultivar used in each field site was used for these comparisons: Russet Norkotah for OR, ID, and CO; Superior for MI, and Russet Burbank for all other sites. Tuber yields of mustard incorporation treatments were compared directly to corresponding non-mustard treatments.

### Soil chemical and biochemical analyses

A suite of chemical analyses were conducted on soils using previously established techniques (Agvise Laboratories, Benson, MN, USA). Organic matter was measured by loss on ignition at 360 °C (Combs & Nathan, 2015), phosphorus by the Bray and Kurtz spectrophotometric method (Bray & Kurtz, 1945; Frank, Beegle & Beegle, 2015), and nitrate-nitrogen by cadmium reduction (Gelderman & Beegle, 2015). Ammonium-nitrogen was measured by gas diffusion and conductivity using a KCl solution (Timberline Instruments, Boulder, CO, USA). Soil pH was measured using a 1:1 soil/water slurry (Peters, Nathan & Laboski, 2015). Bulk soil respiration was measured by infrared laser spectroscopy upon soil rewetting (Gianfrani et al., 2004; Franzluebbers et al., 2000). Total bacterial and fungal biomass, derived from phospholipid fatty acid (PLFA) biomolecular signatures, was measured by Ward Laboratories (Kearney, NE, USA). Soil chemical and PLFA characteristics are shown in Fig. S1, with raw data available in Table S2.

### Variance partitioning analysis of treatments on microbiomes

Statistical analyses were conducted in R v4.3.2 throughout. Analysis of variance tests (ANOVA) were performed in base R. Proportions of variance in microbiome structure attributable to experimental factors and treatments were determined using version 1.32.2 of the variancePartition R package (Hoffman & Schadt, 2016). For datasets of each amplicon in each field site, ASVs present in only 1 of 12 total treatments were omitted, and their counts preserved in a separate column of the count table to maintain compositionality. Count tables were then centered log-ratio (CLR)-transformed. Cultivar, time of sampling (at planting or 60 days after planting), and block number (if applicable) were considered random effects. Fixed effects included rotation length (two levels: two- or three-year) and soil treatment type, wherein logical values corresponded to organic amendment, chemical fumigation, and/or mustard incorporation. The variance attributed to each 16S or ITS ASV from each of these factors was then weighted by ASV mean relative abundance to determine profiles of microbiome-wide variance for each site. ASVs were individually considered to be associated with amended, fumigated, or mustard treatments within particular field sites if the proportion of variance in relative abundance associated with the treatment wa*s* ≥ 0.1. This threshold corresponded to the strongest 2.4% and 3.9% of bacterial and eukaryotic ASV-site-treatment associations, respectively.

### ASV associations with tuber yields

Microbiome samples from each field site, rotation length, and sampling time (8 × 2 × 2 = 32 groups total), were analyzed separately. For each group, reads corresponding to ASVs present in <50% of samples were combined into a single column to preserve compositionality, and counts were then CLR-transformed. Functions within MASS (v.7.3–60.0.1) were used to construct robust linear models of total yields as predicted by CLR-transformed relative abundances of each high-occupancy ASV. Simple linear models were used to obtain summary statistics, including coefficients of determination (R^2^), for each regression. Random subsets of regressions were visually inspected, and ASVs with *p*-values <0.05 and multiple R^2^ value*s* ≥ 0.2 were considered to be significantly associated with tuber yields. Forty-five target ASVs were identified that both (1) increased in abundance in association with a treatment applied at a field site, and (2) were associated either positively or negatively with tuber yields at the same field site.

### Modeling soil treatments, ASVs, and yields

Each management scenario consisted of a soil treatment at a field site under a two- or three-year rotation that corresponded to a control treatment. In the 16 of 33 scenarios where target ASVs were found, we modeled the treatment and CLR-transformed abundance of a target ASV (or the sum abundance of a combination of such ASVs, where multiple were found) against total yields, using multiple linear regressions as shown in Eq. (1).

(1) \begin{eqnarray*}\mathtt{lm(total_yield}\text{ \widetilde }\mathtt{treatment + target_ASVs + cultivar,data)}\end{eqnarray*}

Treatment categories were encoded as dummy variables (0, control; 1, treatment). Different cultivars grown in the same field sites were treated likewise, except in OR and ID data, where only data from the highly predominant Norkotah cultivars were kept. Because ASV associations with tuber yields were specific to the time of soil sampling, models were evaluated for microbiome data collected at timepoints where target ASVs were associated with yields (at planting, or 60 days after planting). Linear models were considered *microbiome-informed* if the increase in abundance of a target ASV (or ASVs) accounted for a significant change in total yield (*p* < 0.05) that was not directly explained by soil treatment. (Follow-up tests confirmed that target ASV abundances increased in response to soil treatments in all cases). To examine more complex interactions between treatments, soil chemical constituents (nitrate, ammonium, and phosphate concentrations, pH, % organic matter), ASV abundances, and tuber yields, we built structural equation models (SEMs) as combinations of linear models using the piecewiseSEM R package v2.3.0 (Lefcheck, 2016) as exemplified in Eq. (2).

(2) \begin{eqnarray*}\begin{array}{@{}ll@{}} \displaystyle &\displaystyle \mathtt{psem(lm(org_matter}\text{ \widetilde }\mathtt{treatment,data),}\\ \displaystyle &\displaystyle \mathtt{lm(target_ASVs}\text{ \widetilde }\mathtt{org_matter + treatment,data),}\\ \displaystyle &\displaystyle \mathtt{lm(total_yield}\text{ \widetilde }\mathtt{target_ASVs,data))} \end{array}\end{eqnarray*}

SEMs were considered *microbiome-informed* if treatments led to increased abundances of a target ASV (or ASVs), which then accounted for a change in total yield that was not directly explained by soil treatment or soil chemical measurements. SEMs were considered valid if *p* > (higher than) 0.05, corresponding to a failure to reject the model. Cultivars and sampling times were handled as described for linear models. For scenarios where multiple models could be built, all combinations were evaluated, and the one with the lowest AIC (Akaike information criterion) was kept. Model statistics for linear models and SEMs are available in Table S3.

## Results

As part of the Potato Soil Health Project, we characterized 1,630 potato soil microbiomes sampled in 2022 from 456 plots spanning 96 discrete agricultural soil treatments implemented in eight field sites across the continental US from Oregon to Maine. This consisted of 858 16S and 772 ITS soil microbiome samples, with mean read depths of 21,162 and 24,116 respectively, and 300,037 16S and 17,214 ITS ASVs in total. Complementary chemical and PLFA analyses revealed differences in soil P, pH, organic matter, and total bacterial and fungal biomass across field sites, and in nitrate throughout the growing season (Fig. S1). We first evaluated the effects of soil management types (organic amendment, fumigation, or mustard incorporation) on tuber yields and on soil bacterial and eukaryotic microbiomes. Next, we identified a subset of amplicon sequence variants (ASVs) associated with tuber yields and specific treatments. From this, we then used analytical models to link soil treatments to ASV-associated changes in tuber yields. Models where treatments changed soil biogeochemistry in ways that influenced yields *independently of the microbiome* are considered beyond the focus of this study.

### Soil treatment effects on tuber yields

Total tuber yields in 2022 varied strongly by field site, rotation length, and their interaction (Fig. 1B, two-way ANOVA field site *F* = 302, *p* < 2e^−16^; rotation length *F* = 9.6, *p* = 0.002; interaction *F* = 5.6, *p* < 4.6e^−6^). Overall, three-year rotations yielded 3.0 Mg/ha (megagrams, or metric tons, per hectare) more than two-year rotations. Across 33 *management scenarios* that evaluated the direct effects of any soil treatment type, organic amendments significantly increased total tuber yields in 4 of 14 cases (29%), and decreased yields in one case. Fumigation showed a similarly modest impact, increasing yields significantly in three of 13 scenarios (23%), with no scenarios decreasing yields. Mustard incorporation, however, did not affect yields in any of six scenarios. Organic amendments in ME1 and fumigations in WI increased yields across both rotation lengths (Fig. 1B), while other interactions between treatments and rotation lengths varied in site-specific ways.

### Soil treatment effects on microbiomes

Using linear mixed model-based variance partitioning, we characterized the effects of soil treatments and other experimental factors (cultivar, rotation length, spatial block within the field, and season in which soils were sampled) on the composition of soil bacterial (16S) and eukaryotic (ITS) microbiomes (Fig. 1C). In sum, all factors respectively accounted for a mean 14% and 28% of the variance in bacterial and eukaryotic microbiomes, respectively. Organic amendment, fumigation, and mustard treatments showed particularly strong effects in some field sites (fumigation in ME1 ITS; mustard in OR ITS; amendment in OR 16S), but not others, and together explained 1.6% more of the variance in the composition of eukaryotic microbiomes as opposed to bacterial ones (*p* = 0.049, *F* = 4.22, one-way ANOVA, type of treatment not significant). Rotation length was the factor that explained the most variance in both bacterial (3.9%) and eukaryotic (11.4%) microbiome composition across field sites, while in contrast, potato cultivar explained the least variance (a mean 0.6%) in the structure of each microbiome type.

We applied the same variance partitioning method to individual ASVs to discern the amount of variance in relative abundance that could be explained by each soil treatment and experimental factor. Noting a sharp decrease in the frequency of variance values above 10%, we defined *treatment-associated ASVs* as those for which any soil treatment explained 10% or more of their variance in relative abundance in all plots and both sampling times within a field site. Only 2.4% of 16S and 3.9% of ITS ASVs were treatment-associated at a particular field site, but 12% of ASVs from both domains were treatment-associated at *any* field site.

**Figure 2:**
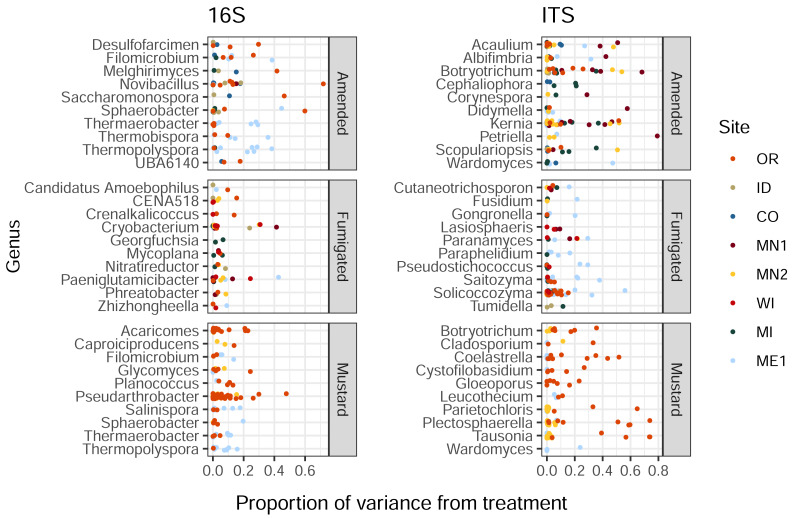
Proportions of variance in ASV relative abundance attributed to either amended, mustard, or fumigated soil treatments, for bacteria (left) and eukaryotes (right). ASVs are grouped by genus, showing the ten genera of each domain with the highest mean variance from each treatment, and which contained at least three ASVs across all field sites. Genera are sorted by alphabetical order.

Organic amendment, fumigation, and mustard incorporation affected soil bacterial and eukaryotic ASVs in different proportions (Kruskal-Wallis *p* < 2e^−16^ and 6e^−5^ respectively). Additionally, these soil treatments imparted a wide range of influence on distinct ASVs belonging to the same bacterial or eukaryotic genus, with less than 1% to over 40% of variance ascribed to treatment (Fig. 2). This trend was also noted within genera from the same field site (in particular, *Solicoccozyma* in ME1 and *Novibacillus* in OR, Fig. 2). These single-site findings suggest that the highly variable impacts of soil treatment on different members of a single microbial genus are not simply a consequence of soil chemistry or climate, and may instead reflect differential responses to selection among species, strains, or ASVs within the same genus.

### ASV associations with tuber yields

Next, we used robust linear regression to associate ASV abundances with total tuber yields at harvest. Yield-associated ASVs, which accounted for 13.2% of bacterial and 11.9% of eukaryotic ASVs, were those whose relative abundances were significantly correlated with tuber yields (either positively or negatively, defined by *p* < 0.05 and R^2^ ≥ 0.2) at either microbiome sampling point during the 2022 season. The number of yield-associated ASVs varied across rotation length, sampling time, and field site, but distributions of R^2^ values did not (Fig. S2).

**Figure 3:**
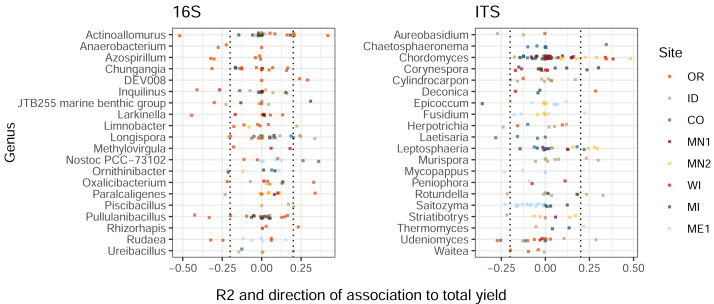
Bacterial and eukaryotic ASVs show variable associations with tuber yields. ASVs are colored by field site. X-axes show multiple R^2^ values of linear models with tuber yields predicted by CLR-transformed ASV abundance, for each combination of field site, rotation length, and sampling time. R^2^ values are multiplied by the sign of the association to yield (positive or negative). ASVs are shown for twenty genera from each amplicon with the highest mean R^2^ values (before accounting for sign of association) and at least three ASVs. Only ASVs present in at least 50% of plots within any field site, rotation length, and sampling time were modeled for relationships to yield. Dotted lines at ± 0.2 indicate correlation cutoffs deemed to be sufficiently yield-associated. Genera are sorted by alphabetical order.

Similarly to the patterns we observed in treatment-ASV associations, ASVs from the same genus displayed widely-varying correlations to total tuber yields (Fig. 3). Among the twenty most highly yield-associated bacterial and eukaryotic genera, most individual ASVs were not strongly positively or negatively associated with yields. Instead, genus-level yield associations were strongly driven by comparatively few outlier ASVs with strong positive and/or negative correlations. Some genera included both yield-positive and yield-negative ASVs, sometimes within the same field site (*e.g.*, *Actinoallomurus*, 16S, OR; *Rotundella*, ITS, ID). Consistent, genus-level directional associations to yields were not found across the continental US, but a few examples within particular field sites included the fungi *Chordomyces* and *Leptosphaeria* (positive in MN2), and the bacterium *Nostoc* (positive in CO; Fig. 3). Some ASVs—in particular, one fungal *Microdochium sp.*—exhibited variable yield associations across field sites, rotations, and sampling times (Fig. S3).

**Figure 4:**
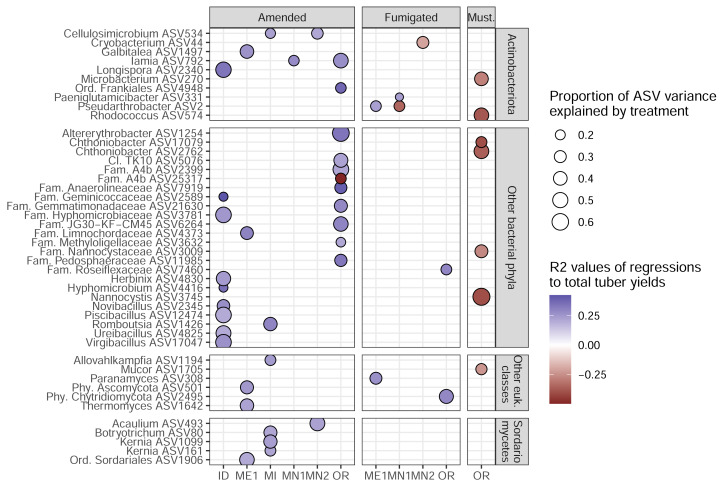
Target ASVs across field sites. All ASVs are enriched in amended, fumigated, or mustard-incorporated soil treatments relative to controls from the same field sites, with bubble sizes corresponding to the proportion of variance in ASV relative abundance explained by the treatment. Bubble colors represent R^2^ values of correlations to total yields; blue positive, red negative. The most specific taxonomic annotations to each ASV are given. Vertical panels are divided by domain (top two, bacteria; bottom two, eukaryotes). All eukaryotes here were classified as fungi except *Allovahlkampfia* (class *Heterolobosea*).

To identify the ASVs most strongly linked to soil treatments and tuber yields, we narrowed our focus onto 45 *target ASVs* (34 bacterial, 11 eukaryotic). These represent the complete set of ASVs that both: (1) increased in relative abundance in a particular management scenario (in which microbiomes from both sampling times were combined); and (2) were significantly correlated, positively or negatively, with tuber yields at either sampling time of any management scenario (Fig. 4). Target ASVs were found in 16 of 33 management scenarios across six of eight field sites, and included several members of the bacterial phylum *Actinobacteriota* and fungal class *Sordariomycetes*. Soil treatment categories were associated with different numbers and yield associations of target ASVs: while organic amendments enriched 34 ASVs, 33 of which were positively associated with tuber yields, five of seven fumigation-enriched ASVs and none of seven mustard-enriched ASVs were yield-positive (Fig. 4). Target ASVs were largely site- and treatment-specific, with only three present in multiple field sites and none associated with more than one treatment category.

### Modeling relationships between treatment, ASVs, soil chemistry, and yields

**Figure 5:**
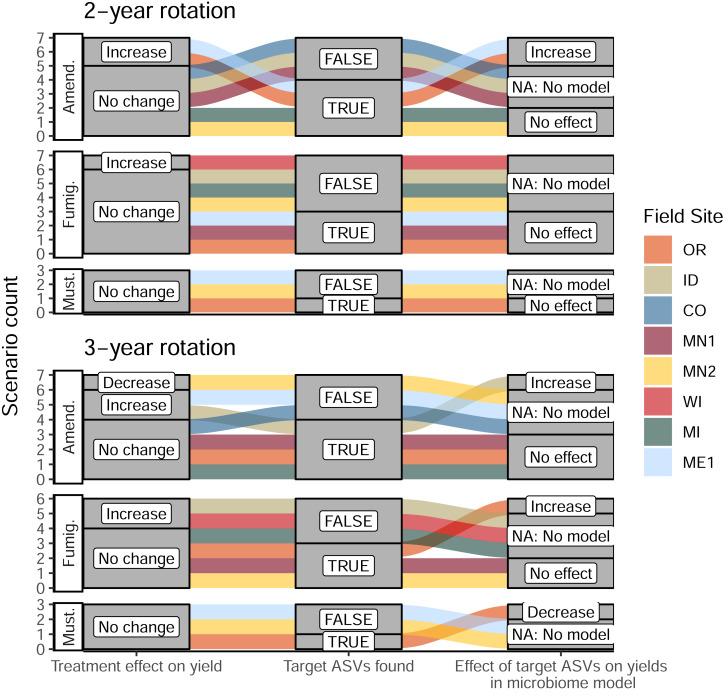
Alluvial plots depicting the detection and contribution of target ASVs towards tuber yields in each management scenario. The left columns indicate whether any significant change in total tuber yield was observed within the dominant cultivar grown at each site, relative to corresponding untreated plots. The middle columns indicate whether target ASVs were detected in bacterial or eukaryotic microbiomes at either timepoint during the 2022 sampling (ASVs shown in Fig. 4). The right column shows whether increased abundances of target ASVs in treatments (relative to non-treatment controls) could explain increases or decreases in total yields within microbiome-informed models. Note that no models could be constructed when no target ASVs were found. Treatments: Amend., Amended; Fumig., Fumigated; Must., Mustard.

Once we identified target ASVs associated with 16 management scenarios across different treatments and field sites, we evaluated the predictive power of their cumulative abundances on total tuber yields at both sampling times during the 2022 season. At the same time, we leveraged a suite of soil biogeochemical data collected across all sites (pH, organic matter, ortho-phosphate, nitrate, ammonium, and bulk respiration) to evaluate whether yield increases could be attributed to changes in soil biogeochemistry associated with soil treatments, independent of shifts in microbiome composition. Among these 16 management scenarios, we identified a total of five *microbiome-informed models* (linear mixed models or structural equation models) in which abundances of target ASVs in bacterial or eukaryotic soil microbiomes could predict changes in yields where soil treatments or biogeochemical parameters could not (Table 1). Target ASVs stimulated by organic amendments were associated with yield increases ranging from 10.4% to 23.2% in three models. In contrast, the only fumigation-based model (implicating one fungal ASV in OR) was associated with a modest 2.6% yield increase, and the only model involving mustard incorporation (on several bacterial ASVs, also in OR) showed a yield decrease of 26.4% (Fig. 5, Table 1, Table S3). Interestingly, these latter two models applied to management scenarios that resulted in no significant changes to tuber yields; in other words, these treatments did not affect yields overall, but nevertheless influenced abundances of yield-associated taxa. These findings suggest the presence of other unidentified microbiome or soil chemical factors that may directly or indirectly counteract the predictive effects of the ASVs in the models. Interpreting these models as “false positives” (because the scenarios did not affect yields overall) suggests that this approach comprehensively identified the ASVs most strongly linked with treatments and yields. In eleven scenarios where target ASVs were identified, links between soil treatment, ASV abundance, and tuber yields were found to be below thresholds of statistical significance. Where no target ASVs were identified, no microbiome-informed models could be built (Fig. 5). Full details of these models are provided in Table S3.

**Table 1:** Management scenarios and model details where target ASV abundances were associated with significant changes in total yields relative to controls. Soil chemical parameters that increased or decreased relative to controls are listed, as well as model statistics and magnitudes of yield changes. Yield increases shown are directly explained by increases in target ASV relative abundances. Full model details are provided in Table S3.

**Field site**	**Rot. length**	**Treatment**	**Trt. effect on yield**	**Soil chem. ↑**	**Soil chem. ↓**	**Model type**	***p*-value**	**R^2^**	**Yield inc. (Mg/ha)**	**% Yield inc.**
ME1	2 yrs	Amended	Increase	None	None	lm	0.001	0.530	3.99	21.3
OR	2 yrs	Amended	Increase	%OM, pH, P, Nitrate-N, P, bulk respiration	NH4-N	lm	0.005	0.728	14.50	23.2
ID	3 yrs	Amended	Increase	%OM, pH, P, bulk respiration	None	SEM	0.275	0.614	6.57	10.4
OR	3 yrs	Fumigated	No effect	None	None	lm	0.013	0.609	1.78	2.6
OR	3 yrs	Mustard	No effect	Nitrate-N	pH	SEM	0.099	0.668	−17.63	−26.4

**Figure 6:**
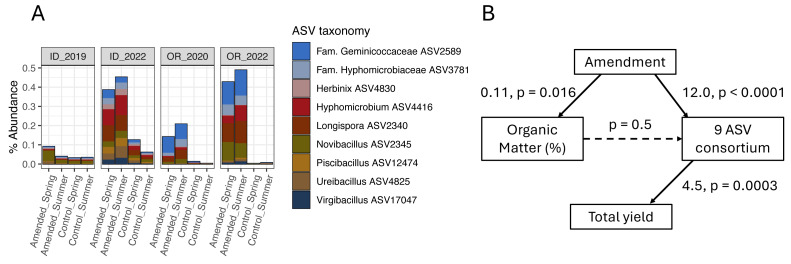
Organic amendments stimulate a consortium of nine bacterial ASVs in OR and ID field sites that are associated with increased yields only in ID. (A) Mean percent abundances of nine amendment-stimulated ASVs within bacterial communities across replicate (*n* = 4) plots in ID and OR field sites in potato growing years (ID 3-yr rotation to 2019 and 2022; OR 2-yr rotation to 2020 and 2022, separated by soil treatment and timing of sampling). (B) Structural equation model (SEM) showing relationships between organic amendment (coded as a binary dummy variable), soil percent organic matter, CLR-transformed sum relative abundances of the nine bacterial ASVs, and total tuber yields in ID 2022 samples (summer 3-year rotations, Norkotah cultivars only, *n* = 14). Model *χ*^2^ = 3.3 with 2 degrees of freedom. For each path within the model, linear estimates and *p*-values are shown, with significant paths represented by solid arrows.

None of the organic amendment-stimulated ASVs from any microbiome model belonged to the same genus, highlighting the narrow taxonomic specificity of these treatment-microbiome-yield relationships. However, microbiome-informed models of amendments in OR and ID field sites were noteworthy in that they linked the organic amendment-associated stimulation of several bacterial target ASVs to increases in tuber yields (a similar model for ME1 implicated two fungal ASVs). While no target ASVs were shared across models for OR and ID sites (Fig. 4, Table S3), the same ASVs were present in soil microbiomes from fields in both states. Compared to plots sampled in 2019 or 2020, all nine target ASVs from the ID 3-yr rotation amendment model were also stimulated by amendments in the OR field site to nearly equal proportions within bacterial microbiomes (Fig. 6A). However, they were not associated with yields in OR. It is interesting to note that while these microbiome shifts accounted for a 10.4% increase in yields in ID, in total they represented only a modest proportion (0.4%) of soil bacterial microbiomes by abundance, even after amendment-associated enrichment.

Two of the three organic amendment scenarios that resulted in yield increases and significant microbiome-informed models also changed soil chemistry: percent organic matter (OM), pH, and ortho-phosphate increased in both ID and OR, while OR soils additionally increased in nitrate and bulk respiration, and decreased in ammonium (Table 1) in response to organic amendment. Additionally, the mustard amendment in OR associated with yield-negative ASVs decreased soil pH and increased nitrate. However, as illustrated in the model of amendment in ID (Fig. 6B), none of the soil biogeochemical changes in any of these models directly influenced ASV abundances. These key taxa may thus operate independently from (or tangentially related to) management-caused changes in soil chemistry.

## Discussion

Amidst a growing recognition of the many ways in which soil microbiomes support plant health and productivity, we leveraged a continental-scale platform consisting of soil chemical, tuber yield, and microbiome data from eight field sites in potato growing regions of the US to investigate how soil management practices used in potato production affect soil microbiomes and tuber yields. Specifically, we hypothesized that (1) soil treatments would affect soil bacterial and eukaryotic microbiomes to different extents across diverse growing regions, and that (2) distinct microbiota impacted by soil treatments could account for increases in tuber yields in field-specific ways. We address these hypotheses after first providing context on how soil treatments affected yields across the continental-scale platform.

### Experimental design, treatment variability, treatment effects on yields

All field sites implemented a four-year rotation with potatoes grown at both two- and three-year intervals, in a factorial design with six discrete treatments. Amounts of total N applied, as well as selection of cultivars, cover crops, and composition of treatments within each field site were chosen to reflect regionally-specific growing practices. Pre-plant soil nitrate and ammonium concentrations varied across sites, but not treatments. Midseason N concentrations did not vary in a treatment-dependent manner, allowing us to discount the effects of N on yields and microbiomes across treatments from the same field site. Other treatment conditions, such as application rates of organic amendments, varied widely, from 2.2 to 34 metric tons/hectare. The treatment with the highest rate (OR) reflected growers with integrated dairy operations that have plentiful access to composted manure; this may be interpreted as an upper bound for realistic amendment rates that influence soil chemistry and microbial composition.

The four scenarios where we observed organic amendment-associated increases in yields all specifically applied composted dairy manure, and at some of the highest rates used across all amendment treatments (≥13 metric tons/hectare, Table S1). Treatment heterogeneity, particularly in the type and amounts of organic amendments applied across field sites, may substantially account for variable influences on yields (Figs. 1B–1C, 5). Different types of organic amendments exert variable, but generally positive, effects on crop yields, and farmyard manure is a particularly effective amendment in this regard (Luo et al., 2018). Variability in soil chemistry, texture, and irrigation status at these field sites, which we observe here (Fig. S1) and have documented previously (Klasek et al., 2023), can also influence the extent to which yield increases are observed with organic matter application (Luo et al., 2018). In particular, high amounts of composted dairy manure added to low-C soils in OR and ID may have been instrumental to yield increases observed at these sites. Regionally variable effects of metam sodium fumigation on tuber yields (Fig. 1B) have also been noted on grower fields across different regions of Wisconsin, highlighting complex relationships with soil texture, chemistry, and microbiome diversity (Shan, Lankau & Ruark, 2024). The effects of specific treatments on soil physiochemical conditions and their relations to yields are clearly complex, and require continued investigation using well-constrained experimental conditions.

The four-year timescale of this study is another important factor when considering that significant changes in yields, soil health, and microbiomes may require consistent, repeated treatments across longer timescales. For example, decadal-scale implementation and monitoring of potato soil treatments has revealed changes in soil chemistry and disease suppression that can take several years to develop (Larkin, 2022). The efficacy of organic amendments on tuber yields did not seem to relate to prior history of potato cultivation, as fields that were both previously potato-naive (OR and CO) and fields that had a history of potato prior to 2019 (all others) each showed mixed results. Crops planted in the year preceding potatoes varied across treatments, but most commonly included wheat, corn, soybeans, barley, clover, various *Brassicaceae*, and mixtures of several crops (Table S1). The complexity of cover crops grown across treatments and field sites precluded straightforward categorization (*e.g.*, in contrast to fumigated *vs.* unfumigated treatments), though cropping history influences soil nutrient availability and tuber production (Blecharczyk et al., 2023), and forthcoming studies will detail these relationships on a site-by-site basis. Nevertheless, this dataset, which consists of 96 specific soil treatments from eight field sites across the continental US (Table S1), provides the most comprehensive and agriculturally-relevant picture of how potato yields and soil microbiomes respond to diverse management practices.

### Treatment effects on microbiomes

Consistent with our first hypothesis, we found that fumigation, organic amendment, and mustard incorporation treatments affected bacterial and eukaryotic soil microbiomes across US potato growing regions in different proportions (Fig. 1C). The variable influences of these treatments on soil microbiomes across field sites likely reflect regional- and continental-scale variation in soil texture, chemistry, and initial microbiomes themselves, particularly at genus and finer-scale taxonomic levels (Klasek et al., 2023). Specific implementations of each treatment, such as the type, amount, frequency, and timing of application likely contribute to their varying influences on soil microbiome composition across field sites. The overall low impact of metam sodium fumigation on soil microbiomes, particularly bacterial ones, may reflect the fact that all fumigation occurred in fall 2021 prior to planting in spring 2022. Metam sodium transiently alters bacterial community composition in the weeks following application, after which the effects of fumigation tend to subside (Castellano-Hinojosa, Boyd & Strauss, 2022; Li et al., 2017). In OR, the strong influence of mustard incorporation on bacterial and eukaryotic microbiomes (1C, 2) may reflect the fact that mustard was planted and tilled into plots every year of the four-year sequence, in contrast to one- or two-year applications at other field sites (Table S1). Soil management, and organic amendments in particular, can influence community assembly patterns of soil microbiota (Neal et al., 2021). Variable effects of organic matter addition on microbiomes may be linked to the complex ways in which they interact with bacterial and fungal phyla relating to soil texture, climate, amendment type, and nutrient stoichiometry (Cui et al., 2023; Delgado-Baquerizo et al., 2017). Considering this, we expect that standardizing the specifics of soil treatments (*e.g.*, types, amounts, and timing of organic matter amendments) across field sites would allow us to better evaluate microbiome responses in soils with varying crop histories and physicochemical characteristics.

In contrast, rotation length was particularly well-constrained within our experimental design: across all eight field sites, 86 of the 96 treatments consisted of pairs of two- and three-year rotations that otherwise varied minimally (Table S1). It is possible that the comparatively high variance attributed to rotation length, particularly for eukaryotic microbiomes, is linked to differences in disease pressures and cropping history between rotation cycles, as noted previously (Qin et al., 2022; Larkin & Brewer, 2020).

### Treatment effects on ASVs, ASVs and yield, and heterogeneity therein

We did not identify consistent correlations between microbial taxa (either bacteria or eukaryotes, at the ASV or genus levels) and soil treatments or tuber yields across regional scales. Instead, our finding that distinct ASVs belonging to the same bacterial or fungal genera were impacted by soil treatments to widely varying degrees (Fig. 2) suggests that closely-related ASVs may respond quite differently to certain soil treatments, even within the same field site. Of course, the compositional structure of microbiome data provides relative, not absolute abundance information. As a result, associations between certain ASVs and treatments or yields that are driven by a perceived increase in abundance may simply reflect decreased relative abundances of other taxa. Differential responses among specific members of soil microbial genera or species may highlight functional diversity as well as ecological and evolutionary processes (Chase, Weihe & Martiny, 2021) capable of supporting strain-level variation (Goyal & Chure, 2025). It is plausible that four years of applied soil treatments, particularly ones that increase soil organic matter or reduce pathogen populations, impart selective pressures among members of microbial populations to drive within-field-site differentiation across treatments, similarly to the differentiation across field sites we previously described (Klasek et al., 2023).

Associations between ASV relative abundances and tuber yields (Fig. 3) can be interpreted in many ways. With this dataset, it is not possible to discern whether these associations reflect direct plant-microbe interactions that promote or inhibit plant growth(such as facilitating plant nutrient uptake, or pathogenicity), indirect interactions (pathogen suppression, or inhibition of plant growth-promoting microbes), unrelated responses to the same stimulus (*e.g.*, organic amendment independently increasing both tuber yields and abundances of certain ASVs), or simply decreased abundances of other (possibly competing) taxa within a compositional data structure. In root-associated bacteria, horizontal gene transfer has distributed genes contributing plant-beneficial functions across wide taxonomic ranges (Bruto et al., 2014; Lemanceau et al., 2017), and recent evidence suggests that key functional traits can play a larger role than taxonomy in predicting plant growth responses (Tran, French & Iyer-Pascuzzi, 2022). Thus, within field sites, genus-level variability in yield associations could be attributed to species- or strain-level differences in functional capacity; across field sites, additional variability may be driven by differences in edaphic or climatic factors, management history, initial microbiome composition (Klasek et al., 2023) and/or heterogeneity within soil treatments. An implication of this variability is that the identification of soil bacterial or fungal populations or functions as potential indicators of soil health and tuber yields may be most likely relevant at regional or local (rather than continental) scales, likely in conjunction with other key contextual information such as soil chemistry, rotation length, or type of amendment.

Within this amplicon dataset, modeling associations at the ASV level was critical for uncovering fine-scale taxonomic variation in treatment- and yield- associations. While this allows for the possibility that a clonal strain with multiple amplicon copy numbers may be represented by multiple distinct ASVs, investigating patterns at this level (as opposed to higher taxonomic categories, *e.g.*, OTU or genus) can enhance predictive power (Song et al., 2025; Wilhelm, van Es & Buckley, 2022), and additionally identify strain-specific responses to treatments (Staley et al., 2018). While metagenomic and/or isolation-based approaches would be required to infer or assess functional diversity at high taxonomic resolution (genus- or species-level), our decision to collect amplicon data allowed us to collect enough samples to investigate relationships across 96 treatment x field site combinations in order to uncover broad patterns linking treatments, microbiomes, and yields.

### Microbiome models

Consistent with our second hypothesis, we identified distinct sets of bacterial and eukaryotic ASVs that were stimulated by soil treatments and associated with increased tuber yields at specific field sites. In four of 33 treatment scenarios, we identified models where soil treatments increased relative abundances of ASVs in ways that explained increased tuber yields relative to non-treatment control plots (Fig. 5, Table S3). Modeling specific treatment scenarios from this continental-scale dataset required subsetting samples by field site, treatment, rotation length, and sampling time, due to the limited occupancy of ASVs across field sites (Klasek et al., 2023) and the variability in ASV-yield associations across time and rotation length (Figs. S2 and S3). While this reduced sample sizes to 12 to 25 measurements (of microbiome taxa abundances, tuber yields, and soil chemical data) per model, the high number of field sites and soil treatments in this dataset allowed us to obtain a robust picture of how commonly-used soil treatments across US potato growing regions affect soil microbiomes and their relationships to tuber yields and soil chemistry.

Compared to fumigation and mustard application, organic amendments were more frequently, and more strongly, linked with microbiome-associated increases in tuber yields, even though they were only effective in three of fourteen scenarios. Inconsistent relationships between fumigation, yields, and soil microbiome characteristics have also been attributed to differences in microbiome diversity or microbial carbon cycling potential across growing sites with distinct soil chemical profiles (Shan, Lankau & Ruark, 2024).

It is surprising that we did not observe more consistent stimulation of microbiota and tuber yields in response to organic amendment. Amendments often increase microbial biomass, soil microbial C/N/P acquisition, and crop yields (Luo et al., 2018; Hartmann & Six, 2023; Moore et al., 2011), and can enrich soil microbial taxa commonly implicated in pathogen suppression (Hemkemeyer et al., 2024; Ashley et al., 2025). It may be that the four-year timescale and/or the variable rates of composted manure applied across field sites were insufficient to reliably stimulate microbially-mediated plant growth promotion or pathogen suppression. One scenario in which organic amendments increased yields in a microbiome-independent manner (ME1, 3-year rotation) could simply be attributed to increased soil nutrient availability for plant uptake. Thus, application of organic matter does not appear to reliably boost tuber yields across growing regions of the continental US, and likely depends on complex interactions between soil physical, chemical, and microbial properties that may occur at local scales.

Inconsistencies between different rotation lengths also puzzled us. Why were we able to build microbiome models for organic amendments in OR (2-yr) and ID (3-yr) rotations, but not vice versa? In our PLFA measurements, we observed that higher microbial biomass and fungi:bacteria ratios in these field sites coincided with rotation lengths where amendments stimulated microbiomes to increase yields (Fig. S4), which may support the idea of a critical soil microbial biomass threshold required for treatments to be effective.

Interestingly, we did not find that soil percent organic matter was linked to abundances of target ASVs in microbiome-informed models for either ID or OR field sites (Fig. 6B, Table S3), which potentially discounts the role of organic matter as a direct nutrient source for enriching target ASVs and stimulating tuber yields. Instead, organic matter application may act to shift nutrient utilization and inhibitory behaviors of key soil taxa (Dundore-Arias et al., 2019). The discrepancy in yield associations in the consortia of organic amendment-enriched bacterial ASVs from ID and OR (Fig. 6A) suggest differences in functional potential across field sites from the same growing region, justifying further efforts to characterize functional variability of plant-health-associated microbiota (particularly as potential inoculants) across agricultural contexts (O’Callaghan, Ballard & Wright, 2022).

### Future directions

While the staggering complexity of soil microbiomes presents challenges for predicting microbial functions from amplicon sequence data (Djemiel et al., 2022), recent efforts have yielded a molecular index predictive of several commonly-used soil health indicators across diverse soil types (Deel, Manter & Moore, 2024). Characterizing specific mechanisms of action by which soil amendments increase abundances of certain taxa, and by which these taxa lead to higher tuber yields, is beyond the scope of this study. But for specific scenarios where these effects were detected, temporally-resolved sampling of soil chemistry, microbial genes and transcripts, and plant and microbial metabolites could be used to establish such relationships (Yu et al., 2021). Linkages between treatment-stimulated soil taxa and endophytic taxa may be of particular importance in potatoes, as machine learning models have recently used endophyte microbiomes to predict tuber yields in subsequent growing seasons (Song et al., 2025). Along these lines, constraining the seasonal variability in treatment-microbiome-yield relationships will be critical to assess viability for grower implementation. In addition, the largely microbiome-independent effects of fumigation on yields call for more thorough mechanistic investigations. While this study provides a crucial baseline for linking management practices to microbiome taxa and tuber yields across the continental US, efforts to integrate large-scale soil health and microbiome data using machine learning approaches are needed to reveal regional- or field-specific targets for managing soil and crop health (Wilhelm, van Es & Buckley, 2022). Finally, exploring the economic tradeoffs growers face while considering soil health improvement strategies allows more insight into how these practices can be more commonly adopted (Maas et al., 2023).

### Conclusions

We gathered a dataset of 1,630 potato soil microbiomes with accompanying plot-level soil chemical data and tuber yields that represent diverse management practices across major growing regions of the continental US. With this, we were able to determine the relative influences of organic amendment, fumigation, and mustard incorporation on tuber yields and microbiome composition, as well as identify microbiome taxa associated with treatments and yields across sites. In four of thirty-three scenarios, modeling efforts identified consortia of microbial sequences that were both stimulated by specific management practices and associated with increases in tuber yields of up to 23% relative to control plots. Three of these four models involved organic amendments, and none were strongly linked with changes in soil chemistry. This study provides a roadmap for identifying specific microbial taxa with growth-characterization potential in association with management practices across US growing regions.

## Supplemental Information

10.7717/peerj.20595/supp-1 Supplemental Information 1 Soil chemical and biochemical characteristics measured across all field sites and sampling times in 2022 Top panel shows soil bacterial and fungal biomass as measured by phospholipid fatty acid (PLFA) analysis, as well as fungal/bacterial biomass ratios. Middle and lower panels show concentrations of ammonium-N, nitrate-N, and P (as measured by the Bray method), as well as percent organic matter, pH, and CO_2 emitted from Solvita bulk soil respiration assays.

10.7717/peerj.20595/supp-2 Supplemental Information 2 Counts and distributions of R2 values of yield-associated bacterial and eukaryotic ASVs across field sites, rotation lengths, and sampling timepoints Numbers of significantly yield-associated bacterial and eukaryotic ASVs across field sites, rotation lengths, and sampling timepoints (top) and distributions of R2 values from each ASV-yield regression (bottom). R2 values are multiplied by the sign of associations to tuber yields. Spring and Summer time points correspond to “at planting” and “60 days after planting”, respectively.

10.7717/peerj.20595/supp-3 Supplemental Information 3 *R*^2^ values of positive and negative ASV-yield regressions across field sites, rotation lengths, and sampling times for fungal ASV68, a Microdochium sp Dotted lines at +/- 0.2 indicate correlation cutoffs deemed to be sufficiently yield-associated. Spring and Summer time points correspond to “at planting” and “60 days after planting”, respectively.

10.7717/peerj.20595/supp-4 Supplemental Information 4 Summer 2022 PLFA measurements of soil bacterial and fungal biomass, and bacterial/fungal biomass ratios in ID, ME1, and OR, by rotation length and treatment

10.7717/peerj.20595/supp-5 Supplemental Information 5 Management details and four years of cropping history for all soil treatments applied at each field site

10.7717/peerj.20595/supp-6 Supplemental Information 6 Soil chemical and PLFA data collected for all plots in 2022, with field site, sampling time, treatment, and cultivar information included

10.7717/peerj.20595/supp-7 Supplemental Information 7 All management scenarios listed by effects of treatment on yield, and whether target ASVs were detected Where relevant, details of microbiome models built from scenarios are provided, along with ASVs and soil chemical data used in modeling.

## Additional Information and Declarations

### Field Study Permissions

The following information was supplied relating to field study approvals (*i.e.*, approving body and any reference numbers): Sampling and publication of results were approved by RD Offutt Farms (Nick David, Fargo, ND), and Miller Research LLC (Jeff Miller, Rupert, Idaho).

### DNA Deposition

The following information was supplied regarding the deposition of DNA sequences: Sequence data is available at the NCBI Short Read Archive (SRA): PRJNA992073.

### Data Availability

The following information was supplied regarding data availability: The code is available at GitHub and Zenodo: - https://sklasek.github.io/potato_soil_treatment_microbiome
- Scott Klasek. (2025). sklasek/potato_soil_treatment_microbiome: Potato soil microbiome analysis repository (v1.0.0). Zenodo. https://doi.org/10.5281/zenodo.16322371.
