# Supplementary figures and images for: Organic amendments to potato soils inconsistently enrich yield-associated soil microbiota across growing regions of the continental US
Source: PeerJ. 2026 Jan 29;14:e20595. doi: 10.7717/peerj.20595 (PMC12861135; doi:10.7717/peerj.20595)

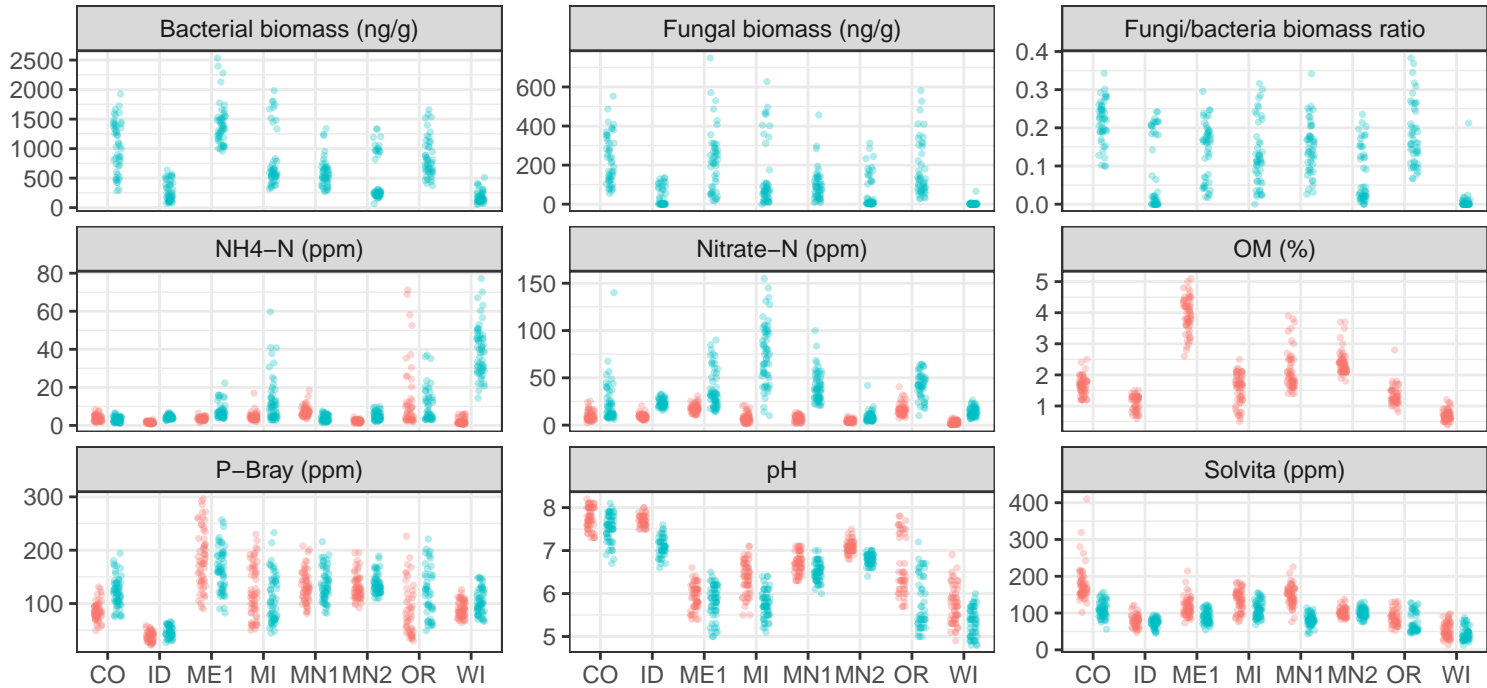

● Pre-planting    ● 60 days after planting

Supplement: Supplemental Information 1 — Top panel shows soil bacterial and fungal biomass as measured by phospholipid fatty acid (PLFA) analysis, as well as fungal/bacterial biomass ratios. Middle and lower panels show concentrations of ammonium-N, nitrate-N, and P (as measured by the Bray method), as well as percent organic matter, pH, and CO_2 emitted from Solvita bulk soil respiration assays. [file peerj-14-20595-s001.pdf]

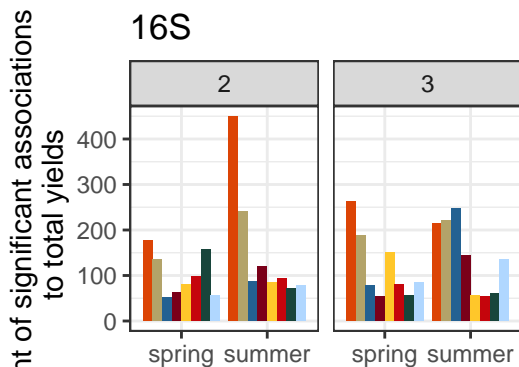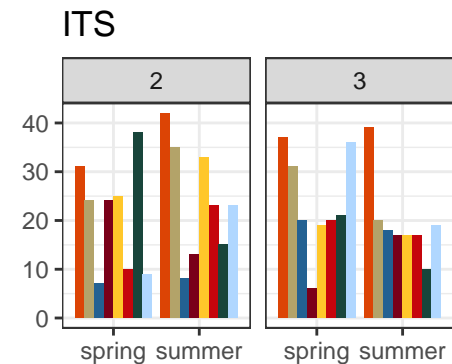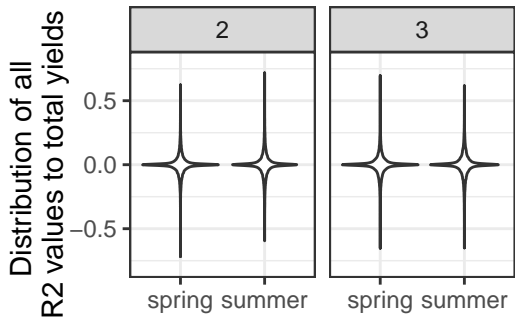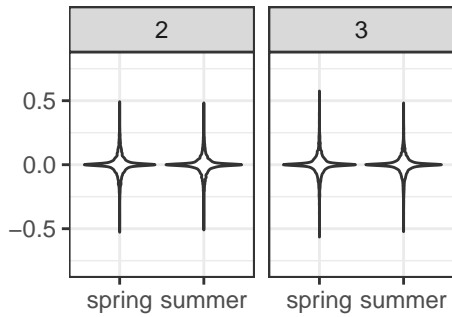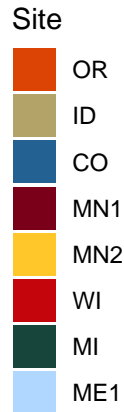

Supplement: Supplemental Information 2 — Numbers of significantly yield-associated bacterial and eukaryotic ASVs across field sites, rotation lengths, and sampling timepoints (top) and distributions of R2 values from each ASV-yield regression (bottom). R2 values are multiplied by the sign of associations to tuber yields. Spring and Summer time points correspond to “at planting” and “60 days after planting”, respectively. [file peerj-14-20595-s002.pdf]

# ASV68, *Microdochium* sp.

R2 and direction of association to total yield

2

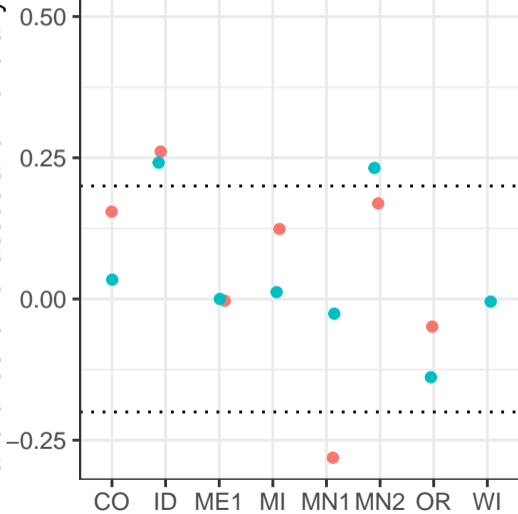

3

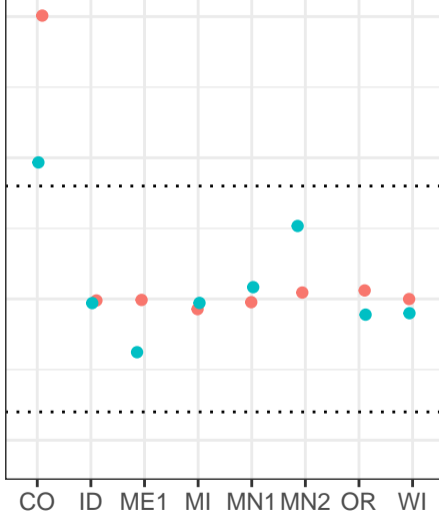

season

spring

summer

Supplement: Supplemental Information 3 — Dotted lines at +/- 0.2 indicate correlation cutoffs deemed to be sufficiently yield-associated. Spring and Summer time points correspond to “at planting” and “60 days after planting”, respectively. [file peerj-14-20595-s003.pdf]

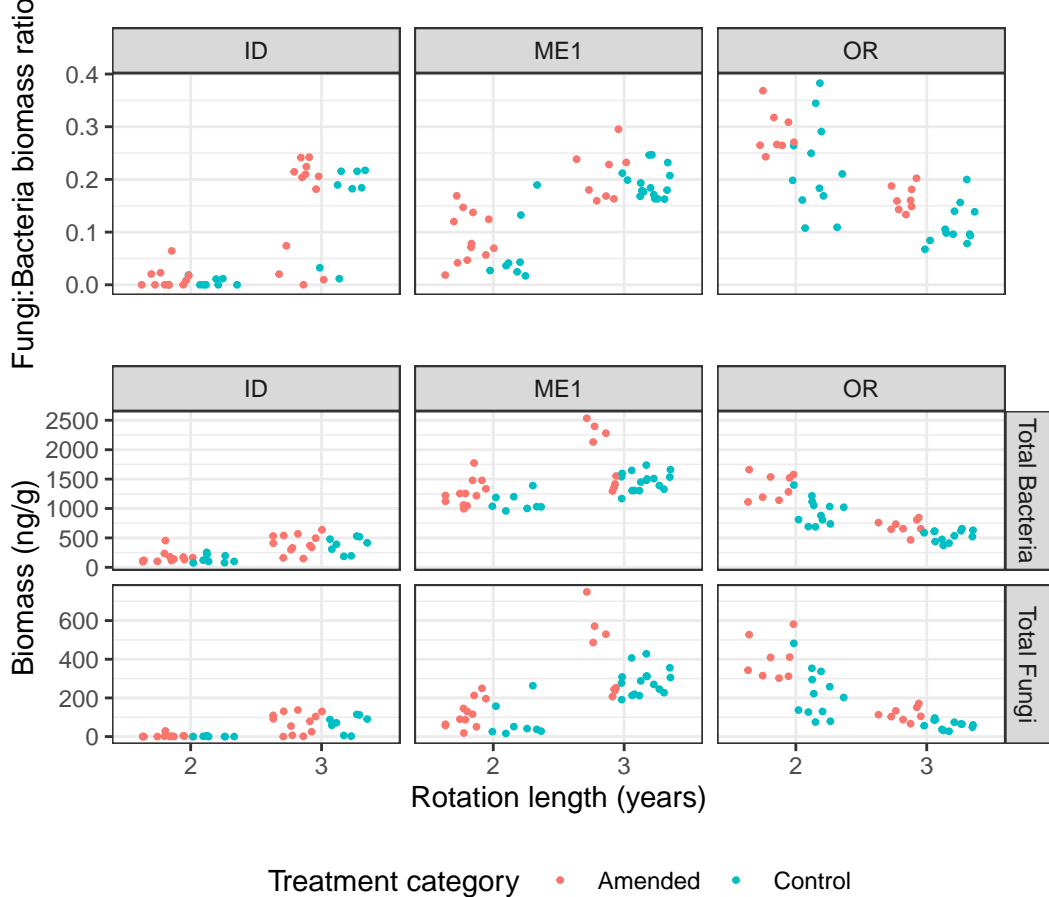

Supplement: Supplemental Information 4 [file peerj-14-20595-s004.pdf]
